# Stigma towards opioid use disorder in primary care remain a barrier to integrating software-based measurement based care

**DOI:** 10.1186/s12888-023-05267-w

**Published:** 2023-10-24

**Authors:** Adriane M. dela Cruz, Tara Karns-Wright, Farra Kahalnik, Robrina Walker, Holly J. Lanham, Jennifer Sharpe Potter, Madhukar H. Trivedi

**Affiliations:** 1 Department of Psychiatry, University of Texas Southwestern Medical Center, 5323 Harry Hines Blvd, Dallas, TX 75390 USA; 2 Department of Psychiatry & Behavioral Sciences, UT Health San Antonio, 7703 Floyd Curl Drive, San Antonio, TX 78229 USA; 3 The Emmes Company, 401 N Washington St, Rockville, MD 20850 USA

**Keywords:** Measurement-based care, Opioid use disorder, Primary care

## Abstract

**Background:**

Opioid use disorder (OUD) is a deadly illness that remains undertreated, despite effective pharmacological treatments. Barriers, such as stigma, treatment affordability, and a lack of training and prescribing within medical practices result in low access to treatment. Software-delivered measurement-based care (MBC) is one way to increase treatment access. MBC uses systematic patient symptom assessments to inform an algorithm to support clinicians at critical decision points.

**Method:**

Focus groups of faculty clinicians (*N* = 33) from 3 clinics were conducted to understand perceptions of OUD diagnosis and treatment and whether a computerized MBC model might assist with diagnosis and treatment. Themes from the transcribed focus groups were identified in two phases: (1) content analysis focused on uncovering general themes; and (2) systematic coding and interpretation of the data.

**Results:**

Analysis revealed six major themes utilized to develop the coding terms: “distinguishing between chronic pain and OUD,” “current practices with patients using prescribed or illicit opioids or other drugs,” “attitudes and mindsets about providing screening or treatment for OUD in your practice,” “perceived resources needed for treating OUD,” “primary care physician role in patient care not specific to OUD,” and “reactions to implementation of proposed clinical decision support tool.”

**Conclusion:**

Results revealed that systemic and attitudinal barriers to screening, diagnosing, and treating OUD continue to persist. Providers tended to view the software-based MBC program favorably, indicating that it may be a solution to increasing accessibility to OUD treatment; however, further interventions to combat stigma would likely be needed prior to implementation of these programs.

**Trial Registration:**

ClinicalTrials.gov; NCT04059016; 16 August 2019; retrospectively registered; https://clinicaltrials.gov/ct2/show/NCT04059016.

## Introduction

Opioid use disorder (OUD) is a serious and deadly illness that remains undertreated in much of the United States [33]. Undertreatment of OUD is particularly concerning, given the strong evidence supporting multiple safe and effective pharmacological agents in treating this condition [25]. However, access to these efficacious treatments remains difficult due to barriers such as stigma, dearth of treatment providers, lack of access due to distance from treatment centers, and treatment affordability [23]; these barriers may be particularly pronounced in rural settings in which barriers to buprenorphine dispensing have been consistently observed [24]. Medical facilities, such as primary care clinics, are positioned to address OUD as they are on the frontlines of the healthcare system and primary care clinics are typically more accessible than specialty addiction treatment settings. Clinicians in these settings also typically have long-term relationships with their patients, creating a system in which patients can be routinely monitored for ongoing symptoms and response to treatment, like management of other chronic medical illnesses (e.g., [11, 13]). Thus, primary care may be one vehicle for increasing access to evidence-based treatments for OUD, as has been previously established [4, 6–11, 16, 26, 32].

Prior work has suggested that many primary care physicians face both systems level [15] and attitudinal [20, 29] barriers in treating patients with OUD with buprenorphine-naloxone. While original work demonstrating these barriers was conducted in the first ten years following FDA approval of buprenorphine, it has not been established if the attitudinal barriers have persisted as a cause for a lack of treatment in the community, particularly following extensive national discussion of the opioid epidemic and requirements by state medical boards for physicians to complete training in opioid-related topics.

Software-delivered measurement-based care (MBC) provides direct support to clinicians and may be one method for overcoming systems barriers to primary care management of OUD with buprenorphine. The term MBC describes the practice of routinely measuring symptoms and side effects of illness and treatment at each treatment visit using itemized symptom rating scales or measurement tools, resulting in more sensitive measures of patients’ status [31]. Patients complete a series of questions on a tablet which informs an algorithm that aids in clinical decisions making. For patients with low technology literacy or questions, someone specifically trained in the use of the tablet is available. The clinician is presented with the results of the patient’s assessments, the accompanying treatment plan suggestions, and potential shared decision-making points (e.g., treatment option discussions, such as the initiation of buprenorphine for the treatment of OUD). We have previously demonstrated that the use of software-delivered MBC for screening, diagnosis, and treatment of major depression significantly improves primary care clinician knowledge and comfort with treating depression as well as patient outcomes [2, 17, 21].

Based on this work, we sought to develop a similar system for the diagnosis and treatment of OUD with buprenorphine. The National Drug Abuse Treatment Clinical Trials Network (CTN) initially funded the CTN-0090 MBC4OUD study as a one-year project to develop the essential components needed to implement measurement-based care (MBC) for OUD utilizing buprenorphine in the primary care setting, including gathering primary care physician and other medical clinician attitudes regarding development and implementation of the project. We have previously published a description of the development of the essential elements of the screening and outcomes assessment tool, including the algorithm for clinical decision support [8].

In this study, we provide a qualitative description of current primary care medical clinician knowledge and attitudes regarding the diagnosis and management of OUD with buprenorphine, including attitudes regarding the use of software-based tools to support this practice. We sought to determine whether primary care clinicians faced barriers that could be adequately addressed via software-based tools for screening, diagnosis, and treatment via clinical decision support or whether the current barriers were beyond those that could be addressed by such a system.

## Method

### Study design

We conducted a qualitative study using focus groups to better understand clinician (MD, DO, advanced practice provider) perception of opioid use and OUD treatment and whether a computerized MBC model could assist clinicians with diagnosis and management of patients with OUD. Focus groups were conducted with outpatient primary care clinicians between January and February 2020 as part of a larger study investigating the feasibility and acceptability of universal screening and MBC for OUD utilizing a software program. We used qualitative analysis to assess clinician perception of opioid use and OUD diagnosis and treatment to better understand the readiness for incorporating universal screening for OUD within outpatient primary care clinics.

### Recruitment

Clinics (19 total) in a north Texas community were contacted about study participation via email. Of the 19 clinics, 7 did not respond, 6 indicated they did not prescribe opioid medications and as a result did not perceive a need for OUD treatment within their clinic and were thus not interested in participating, 3 were not interested in participating and did not give a reason, and 3 agreed to participate. All the non-participating clinics were in a community-based practice setting; all of the clinics that agreed to participate in the focus group sessions were clinics based within an academic medical center.

Participants in the focus groups (n = 33) were office-based primary care physicians and advanced practice providers associated with family medicine, internal medicine, and infectious disease (Table 1); infectious disease clinicians were included as these physicians provide primary care to patients who are HIV + and were thought to have an enriched sample of patients with substance use disorders. Specifically, members of these three departments were invited to attend a focus group session held during their regular department conference time. Participants were informed that the focus group was open to all regular department conference attendees and that the session’s goal was to obtain feedback from medical clinicians regarding current practices for treating patients with OUD and the potential of using computerized MBC for OUD within their clinics. Participants were compensated with lunch. Participants were informed that participation was voluntary and that sessions would be audio recorded. To maintain anonymity, the requirement for informed consent was waived by the Institutional Review Board at UT Southwestern. The protocol and all study procedures were also approved by the UT Southwestern Institutional Review Board and all procedures were conducted following the guidelines and regulations. Data were collected as part of the National Institute on Drug Abuse (NIDA) Drug Abuse Clinical Trials Network (CTN) Study “Innovatively Increasing PCP Prescribing of Buprenorphine: Measurement Based Care and Integrated Electronic Solution (MBC4OUD; CTN-0090).

**Table 1:** *Participant characteristics (N* = 33)

Demographic Variable	*M (SD)* or *N* (%)
Age, *M* (*SD*)	41.24 (11.3)
Race, *N* (%) White Black or African American Asian More than one race Missing	14 (42.42%) 2 (6.06%) 14 (42.42%) 2 (6.06%) 1 (3.03%)
Ethnicity Hispanic or Latino Not Hispanic or Latino Missing	7 (21.21%) 21 (63.64%) 5 (15.15%)
Clinic Role Physician Advanced Practice Partner (Nurse Practitioner, Physician Assistant) Therapist Researcher Missing	27 (81.82%) 3 (9.09%) 1 (3.03%) 1 (3.03%) 1 (3.03%)
Number of Patients Per Week 0–50 51–100 Missing	25 (75.76%) 6 (18.18%) 2 (6.06%)

### Focus groups

The research team conducted three focus groups (one per department) in January and February 2020 (prior to the onset of the COVID-19 pandemic). Focus groups were led by one of the authors (FK) and followed a semi-structured guide prepared by two of the authors (AMD, RW) based on a review of prior literature (e.g., [6, 3, 7, 19], clinical expertise in treating substance use disorders (AMD, RW), and expertise in qualitative data collection and analysis (FK). The guide targeted the following domains: [33] current practices regarding the diagnosis and management of OUD, [25] familiarity and opinions regarding OUD treatment options, [23] positive and negative aspects of offering treatment for OUD in the clinic, and [11] reactions to proposed method of supporting the diagnosis of OUD and treatment of OUD with computerized clinical decision support. These domains represent our goal of differentiating between system-level barriers that could likely be addressed via software-based MBC with clinical decision support and attitudinal barriers that would require different interventions (e.g., education to overcome stigma and misconceptions). Each focus group lasted approximately 60 min. Focus groups were digitally audio recorded and transcribed. Anonymous demographic data (Table 1) were collected at the beginning of each focus group.

### Data analysis

Qualitative analysis focused on identifying themes from the transcribed focus groups. This analysis [12] took place in two phases: (1) content analysis focused on uncovering general themes in the data followed by (2) systematic coding and interpretation of the data.

The first phase of analysis followed an open coding approach [30]. Data coding was first conducted independently by three authors (AMD, RW, FK), a multidisciplinary team of an addiction psychiatrist, clinical psychologist with expertise in substance use disorders, and social worker with training in public health. Data coding began by brief review of the transcripts for the coding team to familiarize themselves with the material. From this review, the coders generated a consensus list of codes and definitions. Each code had a defined list of areas that were included and excluded for inclusion in the code. During analysis, the research team met weekly to discuss coding and preliminary insights emerging from the data. This phase culminated in a list of codes and a codebook with definitions for each code; no subcodes were identified or utilized. Discussion with the full research team led to the final coding scheme.

In the second phase of analysis, the research team systematically coded the data based on the coding scheme developed in phase one [12]. Each member of the coding team (AMD, RW, FK) independently coded each transcript. Discussion was utilized to reach consensus coding of each transcript and discussion and review of coding of each transcript helped to ensure consistent coding across transcripts. A summary report was created to organize data by code across transcripts. Once the data were coded, the research team developed interpretations of the main findings.

## Results

### Participant characteristics

Participant characteristics can be found in Table 1. Most of the participants were white, not Hispanic or Latino, physicians, and saw between 0 and 50 patients per week.

### Main themes

Six major themes emerged from the focus groups and were utilized to develop the coding terms. These themes were “distinguishing between chronic pain and OUD,” “current practices with patients using prescribed or illicit opioids or other drugs,” “attitudes and mindsets about providing screening or treatment for OUD in your practice,” “perceived resources needed for treating OUD,” “primary care physician role in patient care not specific to OUD,” and “reactions to implementation of proposed clinical decision support tool.” Each theme is summarized below and Table 2 provides a list of the themes, definitions of each theme, and a representative example selected by the authors.

**Table 2:** *Major themes, definitions, and examples identified from the data*

Theme	Definition/Description	Example Quotes from the Data
Distinguishing between Chronic Pain and OUD	Providers own perceptions and provider assessment of patient perceptions related to all opioid use: how they define OUD; how they approach patients with pain; how they think about patients with pain or suspected OUD; how they think about OUD; how patients react to being approached; pain management vs. OUD; stigma involved in OUD; how they perceive the need for OUD treatment; how patients’ opioid use (licit or illicit) impacts other aspects of patient’s care.	*“But [discontinuing chronic opioids] is very, very, very hard. I don’t know. I think it’s easier to say that we are better off by not prescribing it from the get-go. I think we are a little stronger about that. But in those who have had the opioids for many years, it’s really hard, really, really hard. You immediately make an enemy, the patient doesn’t wanna talk to you, they go to patient relations, they report you as a bad provider.”* *“What comes to my mind with opioid use disorder? I mean, two things, right? One is the oral, pain medication addiction and opioid use or abuse. And the other one is heroin abuse. I don’t see much of it in my patients; I have two, I think, actively doing heroin. For me, more of the problem is pain medications.”*
Current Practices for Treating OUD	Current practices related to screening, diagnosis, treating, prescribing, etc. in patients prescribed opioids or using illicit drugs, including opioids. Current practice is action regarding patient care (or specific choice of non-action, such as no longer referring to another service) or description of advice/interaction with patient. Includes statements regarding legal or regulatory policy that regulate current practices.	*“So, if you identify the opioid use disorder, as like, the people with heroin, with, you know, that actively use heroin, we tell them about [local treatment program], but they have to want to go. . it’s all voluntary, they have to want to go.”* *“I’ve stopped referring for pain management, ‘cause nobody else will do anything… so I’ve had to learn more and more about opiate medications over the last ten years, just to manage my patients myself”*
Attitudes and Mindsets about Providing Screening or Treatment for OUD	Comfort level, confidence with screening for and treating OUD; attitudes about treatment with buprenorphine or Suboxone; risk vs. benefit of taking on prescribing buprenorphine, including diversion; fear related to prescribing (e.g., patients selling Suboxone on streets); first or secondhand experiences with patients with OUD or SUD; perception of patient’s motivation for care for OUD or SUD	*“I can guarantee you most of them [family medicine clinicians] don’t want to touch this with a ten-foot pole. And it’s not about the training. You know, anybody can go get training online if they want to. It is not about the training. It is about the time. It is about the lack of reimbursement and the lack of resources that has not been addressed.* [Identifier] *here, we’ve had systematic failures, and I feel like we’re adding to that failure by increasing the ability of every Tom, Dick and Harry to prescribe Suboxone* [buprenorphine] *without you know, enough training. Without enough resources. And that is scary. Because pretty soon, yeah, we won’t have opioid use disorder, we’ll have Suboxone* [buprenorphine] *use disorder on top of it.“* *“This [screening and treating OUD in primary care] is super important, but we need resources that I don’t see forthcoming any time soon. . I think we feel like ,. . that patients would have lots of expectations and I think we’re all saying we would have lots of expectations of ourselves to be able to treat the whole person.”*
Perceived Resources Needed for Treating OUD	Any changes in clinic structure or infrastructure needed to support OUD treatment (i.e., hiring and/or training nurse, pharmacist, behavioral health, addressing trauma; adequate referral sources) and need for additional training on any topic (e.g., use of buprenorphine, communication with patients, or on pain management)	*“I don’t think it would make a lot of sense to do the MAT without the integrative behavioral part”* *“There’s [EHR] things that we need help with. There’d be a nurse who could help field the phone calls, ‘cause I don’t know that we could expect all of our nurses to be able to handle those patients. And. . I think it would be super helpful to have a pharmacist who can help us with those titrations”*
Primary Care Role in Caring for Patients	Providers’ understanding of their role as caregivers and their approach to patient care; difficulties and limitation related to medical complexity of patients. Includes expectations providers have for the level of care they give to patients and system demands/expectations or regulatory requirements that must be met for all patients	*“We have [a] complicated patient population that has a lot of different comorbidities, and I think it is us, um, hard to have, um, to try to address a lot of different things in our patient population.”* *“We all have a lot of requirements already placed on us that we’re kind of sort of trying to keep up”*
Reactions to the Proposed Computerized Decision Support Intervention	Anticipated facilitators or barriers to implementation (e.g., impact on workflow, electronic health record integration, time constraints, staff resistance, patient resistance)	*“I don’t think it’s completely impossible, but something has to happen in terms of understanding the dynamics in the clinic and the clinic flow, before anything is instituted. Because any little thing that you add to that routine is gonna seriously impact how we do things.”* *“It sounds helpful. It sounds like a start to some of that infrastructure. . I don’t think it addressed everything, right, but it helps you get started.”*

#### Distinguishing between Chronic Pain and OUD

The focus groups were introduced by the facilitator as an opportunity to explore knowledge and attitudes regarding the diagnosis and treatment of OUD. However, in all focus groups, participants did not respond about OUD specifically but began discussing the difficulties they face in managing chronic opioid therapy in patients with chronic pain. Primary care clinicians in the focus groups reported their patients typically do not perceive chronic opioid use as problematic and reported their patients are hesitant to taper and discontinue chronic opioid treatment. Primary care clinicians reported a desire for training and tools for managing patients with chronic pain treated chronically with opioids, particularly techniques for tapering and discontinuing opioid medications in patients who have been taking these medications for many years. They did not perceive opioid use disorder—often conceived of as heroin use disorder—as a significant problem in their clinical practice, though they acknowledged difficulty in distinguishing chronic opioid use from OUD.

#### Current practices with patients using prescribed or Illicit opioids or other Drugs

When clinicians discussed their current practices for the diagnosis and management of OUD, there was wide consensus that primary care clinicians did not have effective screening tools or processes in place. Primary care clinicians felt that they do not have adequate referral resources for patients with chronic opioid use, whether those are resources for non-opioid pain management or for treatment of OUD. Clinicians in the focus groups expressed frustration regarding the system-level barriers experienced by those seeking OUD treatment.

#### Attitudes and mindsets about providing screening or treatment for OUD

Primary care clinicians reported low comfort level with diagnosing and treating OUD. Clinicians reported a need for additional training for themselves and for their clinical staff prior to starting a buprenorphine treatment program. Clinicians expressed concerns that prescribing buprenorphine may lead to “substitution of one addiction for another”, that buprenorphine would be misused or diverted, and that prescribing buprenorphine without in-clinic services for psychotherapy or case management would not be effective. One clinician expressed concerns that the overall risk/benefit ratio for prescribing buprenorphine in primary care was negative.

#### Perceived resources needed for treating OUD

Clinicians identified the need for additional concrete resources to support prescribing buprenorphine for office-based management of patients with OUD. The following were reported as being needed to manage buprenorphine treatment in primary care: longer clinic visits, nursing staff familiar with buprenorphine, ability for direct support from a pharmacist and behavioral health specialist, communication training (due to concern that typical clinical language would increase shame and stigma), and training in regulatory aspects of buprenorphine and appropriate documentation in the electronic health record. Given this perceived need for additional training of clinicians and staff, some clinicians recommended a potential solution of identifying a subset of clinicians and staff who would manage all patients receiving treatment with buprenorphine.

#### Primary care physician role in caring for patients (not specific to OUD)

An unexpected finding was that, during the focus group discussions, participants spontaneously shared thoughts on their role as primary care clinicians and their relationships with their patients. Many expressed both feeling an overwhelming responsibility to their patients who have complicated, comorbid conditions and a desire to provide high-quality care that meets their patients’ needs. They reported being burdened by increasing demands and expectations placed on them by the healthcare system. Clinicians reported a hesitancy to add diagnosis and management of OUD care to the list of diagnoses they were already managing.

#### Reactions to the proposed computerized decision support intervention

In the final section of each focus group, one of the authors (AMD) provided an overview of the proposed system for software-based universal screening for OUD, utilization of self-report measures for tracking treatment response, and clinical decision support. Focus group participants reported that the proposed set of tools could be helpful, and they expressed concerns about the difficulty of effectively integrating these tools into clinic workflow. They were also concerned that patients would feel burdened by adding additional screening tools and would object to questions about opioid use. Participants were concerned that adding tools for universal screening would not be beneficial without additional resources they perceived as necessary for treating OUD.

## Discussion

This work describes a qualitative analysis of primary care providers’ current practices for diagnosing and treating OUD, their familiarity and opinions regarding OUD treatment options, and their reactions to a proposed method of supporting the diagnosis and treatment of OUD with a software-based clinical decision support tool. This work revealed that systemic and attitudinal barriers to screening, diagnosing, and treating OUD still exist. Specifically, clinicians reported a low comfort level with diagnosing and treating OUD, with fears of inappropriate medication usage, a complicated patient population requiring more care than is available in their clinics, and a lack of referral sources. Notably, the inability to distinguish chronic opioid use from OUD was identified as a significant problem within the clinical practices, though most noted they do not have a current tool in place for screening and diagnosing OUD. Many of the systemic and attitudinal barriers appear to be rooted in stigma against patients with OUD and misconceptions about evidence-based pharmacotherapy for this disorder. Of note, many of the provider concerns regarding “Suboxone addiction” and medication diversion are demonstrably false [27]REF). The knowledge gained from this study indicates that stigma around patients with OUD and evidence-based pharmacotherapy for OUD remain critical barriers to clinician willingness to consider tools for integrating OUD care into their primary care practice.

### Attitudes and mindsets about opioid use and treatment

Stigma associated with OUD is pervasive in the US. The belief that prescribing buprenorphine is simply substituting one addiction for another or that patients will sell or misuse their buprenorphine prescription were highlighted as potential barriers in the current study. Such inaccuratebeliefs [27]REF) are likely reflective of the stigma associated with addiction, even among those who are educated to treat patients with a wide array of medical illnesses. A recent review detailed the existence of stigma between January 1, 1998 to December 31, 2018, finding that significant stigma among providers of medications for OUD was influenced by lack of training and a belief that abstinence is preferred as well as negative perceptions of OUD patients and the associated treatments for OUD [22]. For example, in the current study, one clinician stated, “we’re adding to that failure by increasing the ability of every Tom, Dick, and Harry to prescribe Suboxone without, you know, enough training… Because pretty soon we won’t have OUD, we’ll have Suboxone use disorder.”.” In fact, OUD is one of the only chronic diseases wherein the disease itself *and* the treatment are stigmatized (Note: the authors would like to thank Reviewer 3 for this insightful thought).

To alleviate the impact of stigma related to OUD, and substance use disorders more generally, continued education, training, and policy changes to improve the literacy surrounding the *medical* basis rather than the *moral* basis is necessary [1, 28]. Additionally, addressing the barriers to care that patients experience and improving the healthcare system can help reduce stigma. For example, incorporating behavioral health into primary care and reducing the distinction between mental health and substance use can increase access to care, resulting in a reduction of stigma. Instruction around stigma and OUD in medical education and training is also needed. Furthermore, the screening and assessment of patients’ substance use and accompanying symptoms may alleviate or comfort some patients that may anticipate encountering stigma during a conversation about their substance use.

### Training and practice barriers

Previous literature has highlighted the barriers associated with providing OUD treatment in primary care settings, for example, lack of referral sources and behavioral health support [15, 20, 29]. The capacity of the primary care setting to provide evidence-based treatment for OUD has, historically, limited the implementation of OUD treatment in primary care. The current study indicates that the capacity to provide efficacious OUD treatment in primary care settings is still limited. A theme that was intertwined through our data categories was the perceived complicated nature of patients with OUD. Physicians noted that patients with OUD often have comorbid conditions and previous research has found the complicated social environment that is frequent among patients with OUD can complicate treatment (e.g., homelessness, joblessness, familial stressors, etc.) [5]. However, previous work has also demonstrated that buprenorphine treatment for OUD in primary care is highly effective [32] and that adjunctive psychotherapy provides limited benefits beyond the benefit of medication [34], though a lack of referral options has been cited as a clear issue in referring patients for higher levels of care [18]. It is unclear whether the barriers we identified were related to a lack of knowledge regarding the demonstrated efficacy of buprenorphine in primary care or whether these attitudinal barriers persist despite this knowledge.

### Computerized decision support for OUD screening and treatment

Our project was undertaken with the foundational belief that computerized measurement-based care (MBC) may help alleviate the burden of developing a standard protocol and clinical decision-support tool for treating patients with OUD expressed by the physicians in this study. The participants in the current study expressed that the computerized MBC application could be a helpful tool to incorporate into their practice, though their hesitancy associated with the burden of implementing the application highlights the need for future research to establish efficient implementation protocols. However, this is a small price to pay to develop an implementation protocol that could screen and assist with treatment of patients within primary care - as has been demonstrated in previous research utilizing a computerized MBC application to diagnose and support treatment of depression and anxiety [15, 18].

### Physician identity

Though this analysis revealed some expected barriers – lack of resources (i.e., time, reimbursement) and referral sources and stigma – the existing professional identities of the providers may also play a role in their current lack of acceptance and readiness for OUD screening, diagnosis, and treatment, as unexpectedly revealed during the focus group sessions. Clinicians’ comments coded under the themes of “attitudes and mindsets about providing screening or treatment for OUD in your practice” and “perceived resources needed for treating OUD” highlighted the ways in which clinicians felt they would need significantly more training, skills, and support to provide care to patients with OUD. One potential interpretation of these findings is that the role of the “clinician who treats OUD” falls outside of the professional identity of the primary care clinicians in our sample. Professional identity, which is formed and reified throughout training and practice experience, can have an influential impact on openness to new treatments. As a result, providers feel more closely aligned with institutional norms that are more consistent with their in-group professional identity [14]. However, when outside forces, such as societal changes (in this case, new treatments for OUD) challenge the groupthink and result in institutional changes (for example, reimbursement for SUD treatment), providers may be more likely to hold onto their status quo beliefs and attitudes (in this case, the perceived complicated composition of OUD patients, lack of resources, and stigma).

### Implications

The results of the current study indicate that additional work is needed to adequately and efficiently overcome the perceived barriers associated with diagnosing and treating OUD in primary care settings, most significantly alleviating associated stigma and providing behavioral health support and referral sources. While significant work has been done to reduce stigma and negative perceptions and attitudes associated with problematic opioid use, medical education should continue to be reformed to educate physicians more uniformly on substance use and substance use disorders, with a focus on reducing the prevalent stigma and misconceptions of the treatment of OUD demonstrated by the primary care clinicians in our focus groups. Specifically, continuing medical education for practicing physicians focused on the management and diagnosis of OUD may be informed by interventions that address the role of stigma, attitudes and feelings and physician identity as a barrier to the adaptation of new practice habits, as the clinicians reported seeing care for OUD outside of their identities as primary care clinicians. In addition, research is needed to develop standardized implementation protocols to implement MBC succinctly and efficiently in primary care without disrupting the daily workflow.

### Limitations

Limitations of the current study should be considered when interpreting the results. The participants in the current study are a part of a large academic medical system in Texas. These results may be less generalizable to other primary care practices, especially those that are independently owned or located in more rural areas of the country, or those that have more experience in treating substance use disorders. We also acknowledge that the qualitative nature of this study prevents us from drawing causal conclusions. However, a qualitative study provides us with more details into the attitudes and perceptions of OUD of the participating physicians.

## Conclusion

To increase access to treatment for OUD, existing effective treatments need to be more widely disseminated into community clinics, such as primary care clinics. Software-delivered MBC is one way to aid physicians in treating OUD by providing support at critical decision points with specific guidance on appropriate changes to the treatment plan informed by current symptoms and medication tolerability. This qualitative analysis revealed that while systemic barriers may be alleviated with the implementation of a software-based MBC program, significant work remains to combat the stigma against OUD, as this stigma appears to drive many of the clinicians’ concerns regarding the potential to integrate OUD care into primary care. Further, the software-based MBC program was viewed favorably by the physicians, indicating that it may be a potential solution to increasing accessibility to OUD treatment.

## Data Availability

The datasets used and/or analyzed during the current study available from the corresponding author on reasonable request.
